# Indonesian experts' perspectives on a curriculum for psychologists working in primary health care in Indonesia

**DOI:** 10.1080/21642850.2014.912946

**Published:** 2014-05-05

**Authors:** Diana Setiyawati, Grant Blashki, Ruth Wraith, Erminia Colucci, Harry Minas

**Affiliations:** ^a^ Global and Cultural Mental Health Unit, Centre for Mental Health, Melbourne School of Population and Global Health, The University of Melbourne, Victoria, Australia; ^b^ Faculty of Psychology, Universitas Gadjah Mada, Yogyakarta, Indonesia; ^c^ Nossal Institute for Global Health, Melbourne School of Population and Global Health, The University of Melbourne, Melbourne, Victoria, Australia

**Keywords:** primary mental health care, Indonesia, psychologist, role, skill

## Abstract

Mental health is a critical issue in Indonesia, since its population ranks among the top five in the world and the prevalence of common mental disorders is 11.6% of the adult population. However, the need to build an effective mental health-care system that is accessible to the whole population has only been recently addressed. The Aceh tsunami in 2004 brought to the forefront an unexpected window of opportunity to build a mental health-care system. Integration of mental health care into primary health care is a key strategy to close the treatment gap for people with mental disorders. Existing integration of psychologists into primary health care is a big step to meet the shortage of mental health-care specialists. As primary mental health care is an emerging field, the perspectives of Indonesian experts on Indonesian mental health care are needed to develop a curriculum for training psychologists to work in primary health care. In this study, data have been collected through semi-structured interviews with 24 Indonesian mental health experts, and three focus group discussions with 26 psychologists. Overall, experts agreed that to be able to work in primary health-care psychologists should have roles and training ranging from clinical to advocacy skills. Participants also agreed that psychologists should work in the community and contribute to primary health care as service providers and that strong collaborations between psychologists and other primary health-care providers are the key; these can be developed partly through referral and by respecting each other's unique strengths.

## Introduction

Research in low and middle income countries, including Indonesia, suggests that economic and multi-dimensional shocks, such as illness or crisis, can have an impact on mental health and that this impact is even greater than that from poverty (Das, Do, Friedman, & McKenzie, 2009). Potential disasters due to Indonesia's geographical location, army conflict and economic situation can lead to extreme stressors for the Indonesian population (Bass et al., 2012; Friedman & Thomas, 2008; Souza, Bernatsky, Reyes, & Jong, 2007).

Mental health is a crucial problem in Indonesia. The prevalence of common mental disorders is reported to be 11.6% in the adult population, based on 2007 Basic Health Research (Balitbangkes, 2008). Unfortunately, Indonesian primary health care does not have mental health as a priority. The lack of skills and understanding of mental health problems among existing primary health-care providers means that early detection and treatment do not occur. Mental health hospitals are the treatment option most commonly accessed by the community (Irmansyah, Prasetyo, & Minas, 2009); however, eight of the 34 provinces in Indonesia do not have their own mental health hospitals (Harnowo, 2013). Therefore, in some parts of Indonesia, there is no basic mental health care available and basic, affordable and accessible mental health services are not provided equally across Indonesia. The practice of *pasung* or the physical restraint or confinement of people with mental illnesses (e.g. with a wooden stock, cage, chain or rope) is found all over Indonesia and provides strong evidence that the mental health system is not well prepared to handle mental health problems within the community. It also reflects that there are common misconceptions about mental illness and the healing process in the Indonesian community (Colucci, 2013; Minas & Diatri, 2008; Puteh, Marthoenis, & Minas, 2011; Suryani, Lesmana, & Tiliopoulos, 2011).

The tsunami that hit Aceh in 2004, despite the tragedy, also opened a window of opportunity to strengthen mental health systems locally and nationally (Jones et al., 2007; Prasetiyawan, Viora, Maramis, & Keliat, 2006). Existing primary health-care services were chosen to be the key providers of mental health services. With more than 9000 clinics located all over Indonesia, primary health care was considered a strong component of the health system of Indonesia (Daftar Puskesmas, 2011). Every primary health-care clinic serves approximately 30,000 people. The average staff number of each existing primary health-care clinic is 30 people with various professional backgrounds, including doctors, dentists, nurses, midwives, sanitarians and administrative staff (Abdullah, Hort, Abidin, & Amin, 2012).

Each primary health-care clinic has six priorities, yet none of these focuses specifically on mental health. These priorities are: (1) maternal, new-born and child health programmes, and family planning; (2) community health nutrition programmes; (3) health promotion; (4) basic sanitation and environmental health; (5) immunisation and control of communicable disease and (6) common illnesses treatment (Abdullah et al., 2012). Task-shifting to non-mental health specialists has been tried in Aceh by training existing medical doctors or general practitioners (GPs) to be what have been termed, ‘GPs+’, by equipping them with mental health training. Several nurses were also trained to be community mental health nurses (Prasetiyawan et al., 2006). This was a good first step that could potentially be duplicated in other parts of Indonesia.

At the same time, it has been argued that there is a pressing need to integrate mental health specialists into the workforce of existing primary health-care providers (Eaton, 2012). Evidence has demonstrated that the most effective and cost-effective way to treat most mental illnesses is by combining pharmacotherapy and psychotherapy, rather than pharmacotherapy or psychotherapy alone (Patel et al., 2007; Patel, Koschorke, & Prince, 2011). This raises the idea that Indonesia's psychologist workforce who can deliver a range of psychological treatments as part of their basic skills would be the best-suited mental health specialists to be integrated into the existing primary health-care settings to treat mental illness in collaboration with GPs.

The comorbidity between chronic illness and mental health problems is also a well-recognised phenomenon (Cohen, 2008; Craven & Bland, 2013; Elser, Weisberg, Sciamanna, & Bock, 2004; Fisher, Chan, Nan, Sartorius, & Oldenburg, 2012; Sanna et al., 2013). Indeed, research has found that health-care costs increase significantly for those patients with chronic illness who have physical comorbidity combined with mental illness (Jones, 2013). Furthermore, it seems that the separation or fragmentation of health care for such patients tends to lead to higher costs and on average poorer clinical outcomes (Laderman & Mate, 2014).

In contrast, the integration of mental health interventions into primary health settings has been found to be associated with reduced long-term costs, better care and fewer re-admissions to hospital (Laderman & Mate, 2014; Morrissey, 2013). For example, an evaluation of a 12-month integration of psychologists into two family practices in Eastern Ontario, Canada, showed not only were patients and doctor more satisfied, but the change also resulted in better clinical outcomes and reduced mental health billing by the doctor (Chomienne et al., 2011).

There are two common models of integration of psychologists into primary health care. The first is *colocation*, where psychologists work together with doctors, nurses and other health professionals in one primary health-care clinic and is a model that has been outlined by Searight (2010) in his book *Practicing psychology in primary care*. One example of this model is practised in Veterans Affairs Medical Centres Primary Care in the USA. Psychologists and primary health-care providers work together to provide multidisciplinary care (Elder & Silvers, 2009). The second model of integration of psychologists into primary health care is often termed *shared care* and involves psychologist continuing to work in a separate location, usually private, but nevertheless working closely with primary health-care providers through close referral networks and inter-professional communication. This model is common in Australia and the Netherlands (Derksen, 2009; Moulding et al., 2007).

Turning to the Indonesian context, the field of psychology has a substantial workforce – Himpunan Psikologi Indonesia (HIMPSI) or the Indonesian Psychological Association has around 9100 members and the number of university faculties of psychology across Indonesia is 82 (HIMPSI, 2012). However, most psychologists are working in private practice, which makes their services unaffordable for a large proportion of the population. Therefore, integrating psychologists into primary health care in Indonesia may be one good strategy to provide mental health care, which is accessible to a wider range of the population of varying economic levels.

In 2004, a pilot project of integrating psychologists into primary health care in Indonesia was undertaken by the Sleman District (a district within Yogyakarta Province) in collaboration with the Faculty of Psychology, Universitas Gadjah Mada. The Local District Government funded the project. Starting in 2004 with only six psychologists colocated in primary health-care clinics, the project has since expanded to 25 psychologists, which in practice means that there is one psychologist allocated for each primary health-care clinic. Since July 2010, Yogyakarta Municipality (under Yogyakarta Province) has also commenced the same programme, with at the last report, one psychologist is available for two primary health-care clinics (Retnowati, 2011).

According to Retnowati (2011), in an evaluation of the Sleman district pilot project it appeared that the psychologists were faced with some particular difficulties while working in the primary health-care setting, such as not being skilled to deliver short interventions that are more appropriate to the constraints and culture of primary health care. Indeed, in this same project, it was reported that the other primary health-care providers also experienced some confusion about the role and skills of psychologists. Thus, the curriculum of the psychologists' training did not necessarily prepare them to work in this setting.

It is useful then to reflect on the training of Indonesian psychologists during their required six years of training. It involves a four-year undergraduate degree and a two-year Masters programme, which includes a supervised internship. HIMPSI or the Indonesian Psychology Association is the registration body (HIMPSI, 2001, 2003) that oversees the training, and in the two years of Masters training students are trained as specialists, choosing an area of expertise, such as clinical psychology, educational psychology or industrial/organisational psychology. Of note, although they are trained to deliver specialist treatments they are poorly prepared to work within the primary health-care setting (Retnowati, 2011). In part, this may be because the current curriculum of clinical psychology education throughout Indonesia is dominated by assessment and intervention skills (Universitas Gadjah Mada [UGM], 2006).

As an emerging field, to date there is no literature that can specially inform the development of a core curriculum for psychologists working in an Indonesian primary health-care setting. Therefore, the aim of this study was to investigate the roles and skills needed by psychologists to work in Indonesian primary health care and inform the development of a curriculum. The specific research questions addressed within this study were: what are appropriate *roles*, central *skills*, and the academic and clinical *training* requirements of psychologists to work in primary health care, according to experts in Indonesian mental health care?

## Methods

### Design

To gain a comprehensive understanding of Indonesian experts' perspectives on the roles, skills and training requirements for psychologists working in primary health care, semi-structured interviews and focus group discussions were conducted. The semi-structured interviews involved 24 Indonesian experts from various backgrounds and the three focus group discussions involved 26 psychologists currently working in primary health-care clinics.

#### Semi-structured interview

The conduct of the semi-structured interview was informed by current best practice recommendations. As outlined by Rabionet (2011), the semi-structured interview is a research tool that allows participants to tell their own stories within a flexible interview guide and has the advantages of enabling the interviewer to probe the responses that contain any ambiguities, and clarify information that is too complicated. Notably, this type of interview is designed with structured questions without response codes, which allows flexibility and open mindedness (Bowling, 2009). This method has been used extensively in curriculum development and in the health area. The interviews were conducted with a keen awareness of the pitfalls such as being time-consuming for participants and the potential risk of interview bias (Cavkaytar, Ceyhan, Adiguzel, Uysal, & Garan, 2012; Mackinlay, Jelinek, & Weiland, 2011; Yurekli, 2012).

#### Focus group discussion

Similarly the approach to the focus groups was informed by current best practice in the literature. As Kitzinger and colleague (Kitzinger & Barbour, 1999) have noted, one of the key differences between focus groups and other group interviews is that focus group's ultimate aim to generate data. One advantage of this approach is that when conducted carefully, it creates an environment that helps participants to feel safe to discuss a topic that may be sensitive, embarrassing or difficult to discuss in the personal interview (Bowling, 2009). Therefore, this technique was chosen for interviewing psychologists in this study because an exploration of lack of competence in appropriate skills for working in a primary care setting may be an embarrassing subject. Our intention was that through the focus group discussion, participants would discuss their ideas freely and be stimulated by the group dynamics. A formalised interview guide was produced for use by the focus group facilitators.

### Participants

The participants for the semi-structured interviews were experts from a variety of disciplines in Indonesian mental health care, and especially those involved in the integration of psychologists into primary health care. Twenty-four experts from local and national levels were interviewed. For the focus group discussions, 26 psychologists working in primary health care as part of the pilot project in Sleman District and Yogyakarta Municipality were interviewed.

Participants were invited through the following methods:

1. Publication: The researcher reviewed the Indonesian mental health-care literature and identified author names that were frequently published or cited by others. In practice, most of these published papers in this niche field were found in the *International Journal of Mental Health Systems*. The researcher contacted the nominated expert to offer voluntary participation in the semi-structured interview.
2. Snowball sampling: Many experts were also identified through the networks of the Centre for Public Mental Health, Universitas Gadjah Mada, Indonesia. After obtaining agreement from a participant, the researcher asked for recommendations for other opinion leaders and experts.
3. Recommendations from key professional organisations: The researcher asked for recommendations from two keys professional organisations involved in the process of integration of psychologists into primary health care: the Indonesian Psychological Society (HIMPSI) and the Clinical Psychology Association (IPK).

A formal invitation letter, a plain-language statement and consent form were sent through email to nominated participants. In the plain-language statement, there was an explanation of the aims of the interview, the way the interviews would be conducted, how confidentiality would be maintained and an explanation that the interview would last for one hour. The email invitation was then followed-up by a phone call or text message (SMS) from the researcher (DS). Except for three nominated experts, all the invited participants agreed to participate. Twenty-four experts agreed to be interviewed and 26 psychologists currently working in primary health care agreed to participate in the focus group discussions. Table 1 presents the characteristics of the interview and focus group discussion participants.

**Table 1.** Characteristics of participants.

Attribute	Frequency	Percentage
Interview		
Working level (national or local )		
National	10	41.7
Local	14	58.3
Gender		
Male	8	33.3
Female	16	66.7
Profession/role		
Psychologist working in primary health care	2	8.3
Psychiatrist	4	16.7
Medical doctor	1	4.2
Community Mental Health Nurse	1	4.2
Professional Association Representative	2	8.3
Dentist	1	4.2
Policy-maker	2	8.3
Lecturer/University	2	8.3
Midwife	1	4.2
Health Officer	5	20.8
Cross Cultural Experts	1	4.2
Public Health Practitioner	2	8.4
International publication in Indonesian mental health care		
Yes	5	20.8
No	19	79.2
Focus group discussion		
Working level (national or local )		
Local	26	100
National	0	0
Gender		
Male	0	0
Female	26	100
Profession/role		
Psychologist working in primary health care	26	100
International publication in Indonesian mental health care		
Yes	0	0
No	26	100

### Procedure

The semi-structured interview and focus group discussion guides were developed based on the research questions. The guides were then piloted with three Indonesian psychologists. Based on their input, the final interview guide was developed. The questions in the guide were about the roles, skills and training of psychologists working in primary health-care settings. Table 2 presents the key questions included in the semi-structured interviews and focus group discussion.

**Table 2.** Interview guide.

Aim	Questions
To assist in increased knowledge of training for psychologists working in primary health care and to encourage knowledge sharing across groups	(1) What are appropriate *roles* of psychologists working in primary health care?
	(2) What are the key principles for psychologists to be able to collaborate with other professionals in primary care?
	(3) What are the barriers for psychologists working in primary care?
	(4) What are the central *skills* of psychologists working in primary care?
	(5) What are the academic and clinical *training* requirements for psychologists working in primary care?
	(6) What is the current academic and clinical training curriculum in Indonesia?
	(7) What aspects of the current academic and clinical training curriculum need to be improved?

Semi-structured interviews were conducted, in Jakarta or Yogyakarta. At the agreed time and place, one of the authors (DS) conducted and recorded all the interviews.

The focus group discussions were conducted at the Faculty of Psychology, Universitas Gadjah Mada, Yogyakarta. The 26 participants were divided into three groups; one group included those who were trained as psychologists before the year 2000, the other two included those who were trained as psychologists in the year 2000 onwards. The facilitator of the group receiving training before the year 2000 was DS, while the other two groups were facilitated by two alumni of the Faculty of Psychology, Universitas Gadjah Mada, who had already being selected and trained by DS. All the focus group discussions were recorded.

### Data analysis

The data collected was analysed using a thematic analysis approach, which was informed by four key methodological concepts that have been described in the literature; *theoretical rigour*, *procedural rigour*, *interpretative rigour* and *rigorous reflexivity*.

First, *theoretical rigour* is the sound reasoning used to conclude that the method is appropriate to answer the research problem, and in the case of this study thematic analysis was chosen. It is a qualitative data analysis method, which involves the identification of codes from the data inductively (Rice & Ezzy, 1999) and it was chosen because it requires a lower level of interpretation than other analytical approaches, such as grounded theory or hermeneutic phenomenology (Sandelowski & Barroso, 2003). Since this research was mainly about experts' perspectives based on their opinion and experience, a low level of interpretation was needed. In thematic analysis, the focus is on the context and integration of manifest and latent content, and not the frequency of the themes (Vaismoradi, Turunen, & Bondas, 2013). The thematic analysis involves the search for common threads that arise across the interview (DeSantis & Ugarriza, 2000).

Second, in undertaking this study the researchers were mindful of *procedural rigour*, an approach that refers to the transparent documentation of methodological and analytical decisions. This first step consisted of transcribing the interviews and reading the data more than once to get initial impressions. The next step was to develop initial codes by finding relevant and interesting facts in the entire data. Generating themes was the third step, and was carried out by searching for potential themes within the codes. Then, the last step was extracting and organising the final data to answer the research questions (Braun & Clarke, 2006). NVivo10 software was employed to manage the data and generate the coding in this research (QSR International, 2012).

Third, *interpretative rigour* involved in particular careful attention to checking for consistency. In practice, DS shared the data with the research team to check for consistency before deciding on the final thematic categorisations. Intensive discussion was conducted within the research team.

Finally, rigorous reflexivity (Rice & Ezzy, 1999), another important concept described in this methodological approach encouraged reflection about the honesty of the researcher's roles and the authenticity of the data and its reporting.

## Results

The results are presented below. The source of the data is displayed in a bracket behind each of the quotes with ‘FGD’ standing for focus groups discussion and ‘P’ referring to a participant in a semi-structured interview. FGD-1, for instance stands for ‘focus group discussion, group 1’, FGD-2 indicates that the resource is group 2. The data that come from semi-structured interviews are displayed as P-1, P-2 and so on, with the P standing for ‘participant’ and 1 representing the number assigned to this subject in the data coding.

### Important roles and skills of psychologists in relation to patients

Most participants agreed that psychologists could have a wide range of roles in primary health care, ranging from health promotion to rehabilitation. The basic roles and skills in primary health care according to one focus group member (FGD-1) was ‘providing clinical services and community services’. However, one expert pointed out in an interview that the range of services should also include ‘promotive, preventive, curative, and rehabilitative. The programmes include general clinics, family planning, mother and child health, environment sanitations and so on’ (P-1). For example of a promotion programme, one FGD participant explained that psychologists should ‘promote good nutrition, healthy pregnancy’ (FGD-1). Another FGD participant stated that psychologists' role should include ‘prevention of psychosocial problems in the society, such as anxiety and free sex’ (FGD-2).

One participant took the view that there are three main types of patients in primary health care, and psychologists can have different roles with each of these types of patients, ‘healthy patients, high-risk patients and patients with mental health problems’ (P-17).

There was a consistent recognition that psychologists ought to be very skilled in conducting assessments. According to one interview participant, conducting ‘assessment’ (P-6) is an essential skill of psychologists. In line with this, another participant explained that a psychologist should have ‘skills to assess the problem of patients’ (P-11). Another participant pointed out that ‘the assessment should be brief and not too long’ (P-2).

There was also a strong recognition that psychologists ought to be able to provide psychological treatments for mental health problems, and discussion about how this might fit into primary care settings. One participant explained that the psychotherapy ‘should be brief. If we have skills to deliver therapy that requires a long time, this will not match with the primary health-care context. The therapy should not have so many sessions. It should be brief’ (FGD-3). Another participant pointed out that the role of psychologists is to provide a non-pharmacotherapy treatment: ‘psychologists do not use medicine. Pharmacotherapy is actually only 30%, the other 60% is psychology, cognitive and so on. In primary health care, it is something that is overlooked, because nobody is in charge on it’ (P-2).

The participants also highlighted that the therapy should be evidence-based. ‘The therapy in here is not merely counselling. Counselling is one basic technique, but we need more than that. We need cognitive approaches, and other evidence-based therapies’ (P-8).

### Important roles and skills of psychologists in relation to other primary health-care providers

Most of the participants agreed that psychologists should acknowledge other disciplines. For instance, one participant explained, ‘psychologists should have a broad understanding on mental illness. With the development of psycho-neurology and psychopharmacology, psychologists should be open-minded. They cannot think that psychology is the single best approach’ (P-9). Another participant pointed out that to be able to acknowledge other health professionals, psychologists should:

 … understand their own limitations. It is the basis of collaboration. We should acknowledge the other professionals' skills and understand our own limitations. So psychologists will understand that mental illness is not only the problem of clinical psychologists. Mental illness needs to be handled by multi-professionals and we need to provide comprehensive services. (P-8)

Psychologists should also be capable of providing education about mental health to other health professionals, as highlighted by one participant:

I would say working closely with a team which focuses on problems of the elderly, and teaching them what the major mental illnesses, and how to recognise them and what to do about it, should be one of the roles of the psychologists. And then, what about maternal child health? They really need to teach the nurses who are seeing all the pregnant mothers and all of the new mothers how to recognise post pregnant depression, right? They need to know that. And mostly it's not treated, right? And psychologists also need to give a good training to people about developmental problems. (P-23)

Psychologists should be trained in basic medicine, as highlighted by one FGD participant:

In academic training, psychologists need to be trained with regards to the common diseases in primary health care. For example, a session from dentists about the process of dental therapy and the anxiety of patients to meet dentists. Psychologists can give interventions in this area. (P-1)

Another theme was the idea that psychologists working in primary care should have a basic understanding of physical conditions and their treatment . One FGD stated, ‘psychologists should understand the differences between gastritis, chronic and acute, how much medicine does someone take and what are the psychological-related conditions. Psychologists also should understand about hypertension and diabetes’ (FGD-4). Participants also stated that psychologists should understand diagnosis and medication in order to contribute to a primary health-care team. Knowledge regarding medication should include how the medicine works, the side effects and why people stop taking medicine, as highlighted by one participant.

In the PUSKESMAS [primary health care] there are constant misdiagnoses and there is a huge problem of mistaken medications. So I would say, given my view, number one you need skills to diagnose, number two you really need to understand medication even though you don't do the prescription, but you need to tell, you need to work with the doctor and say ‘this person in my view needs this medication’, etc. You need to understand why people stop taking the medication. You need to understand what are the side effects and why choose this medication, what are the patients' right? (P-23)

In line with the above opinion, another participant pointed out the importance of pharmacology to be able to collaborate with other health professionals:

Patients in primary health care do not care which health professional [is] giving service to them. They would ask about medicine and the side effects to any health professional. If psychologists don't understand, it will make primary health care not a one-stop service. Patients need another queue just to see the GP to ask about medicine information. (P-2)

The FGD participants reported that the collaborative team in primary health care should not only consist of doctors, but all of the elements in primary health care, namely nurses, midwives, nutritionists and dentists. ‘Our relationship with them will influence their willingness to refer patients to us. For the time being, we get referrals mostly from doctors, nutritionist and midwives’ (FGD-5). Another FGD participant added, ‘The key is on the doctors, if doctors can explain to patients why they are referred to a psychologist, patients will love to be referred’ (FGD-6). Participants highlighted that ‘a good relationship with the primary health-care team is a key. Most of the patients will come to see doctors or midwives, not psychologists’ (FGD-7).

Other participants reported that home-visits with other health professionals to patients' houses are another important part of collaboration with other health professionals. They reiterated that psychologists need to visit and educate patients who ‘have chronic diseases, who do not want to take medicine’ (P-11). Another participant added, ‘I used to do home visits with other health professionals, even though sometimes my role is not dominant in the home visit’ (P-18).

### Important roles and skills of psychologists in relation to primary care as service providers

Most of participants reported that in the context of primary health care as a service provider, psychologists should be involved in designing a mental health programme. A participant said, ‘It is very important for psychologists to develop a good mental health program, step-by-step’ (P-2). To be able to do this, psychologists should have a good understanding of the demographic conditions of the people attending the primary health-care clinic where the psychologists work. As one participant highlighted, ‘They have to know the condition, geographical condition, population, statistical condition of elderly and children. So they can design tailored interventions or programs’ (P-5).

Participants also reported that psychologists need to understand the success indicators of primary health care that have been set nationally. One participant explained, ‘the indicator, such as coverage, utilisation. We also have to measure the output of our services with specific instruments that we have prepared’ (P-2). Participants also stated that psychologists need to undertake an ‘evaluation of services and research’ (P-3) and should also understand organisational management from ‘planning, organising, implementation and evaluation’ (P-13). Creating a customer satisfaction survey is also part of an evaluation, as explained by one participant: ‘In PUSKESMAS Turi, psychologists help to make a customer satisfaction survey. We should make this survey every year and it was very good when psychologists were in charge in it’ (P-12).

Psychologists also need to contribute to build a referral system in primary health care. As a participant explained: ‘We should know how to make referral systems work, and we should initiate good collaborations with insurance companies’ (P-13).

### Important roles and skills of psychologists in relation to community

Participants reported that in primary health care, psychologists work closely with communities: ‘We work with communities, not being exclusive. If there are diseases that are related to psychology, we need to give psycho-education directly to the community’ (FGD-7). Other participants reported that in the community, psychologists deliver ‘psycho-education’ (P-6) and family education to ‘patients with severe mental illness, the family and carer need to be educated by psychologists’ (P-18). Therefore, public communication skills and social skills need to be mastered by a psychologist and ‘psychologists need to be ready to talk in front of the public at anytime’ (P-18).

Another participant pointed out that managing stigma is one of the psychologist's challenges, ‘in the mother and child health section, we refer patients to psychologists, but psychologists means mental health problems, so some patients are afraid to be referred to psychologists’ (P-3). Therefore, psychologists need to promote their own roles: ‘currently, I talk to the community more about positive psychology, not merely about anxiety. I hope the community will perceive that psychologists can handle more than just negative problems’ (P-18).

Another participant suggested that contribution to public health through ‘community health programmes’ (P-14) is one of the essential roles in primary health care. Therefore, ‘basic knowledge of epidemiology’ (P-4) is also essential. It was also suggested that psychologists need to understand many facilities that exist in the community and refer patients to them if necessary. In this context, psychologists need to have skills to ‘negotiate with multi-sectoral agencies’ (P-14).

Since Indonesia is a disaster-prone country, psychologists are expected to handle ‘disaster recovery’ (P-9): ‘Psychologists in here need to handle disaster recovery of Merapi mountain eruption’ (P-12). In this situation, crisis intervention skills and understanding of psychological conditions of the community after disasters are needed.

### Important roles and skills of psychologists in relation to policy

In relation to policy, participants highlighted that psychologists need ‘to understand health systems’ (P-4). Another participant expected that psychologists would be able to ‘advocate not only in local level, but in national level’ (P-9). In line with this expectation, another participant added, ‘I think some policy-makers are resistant to receive psychologists as a new solution. We need to do something to prove that it is a real expectation from the community’ (P-6). Participants also pointed out that to have strong advocacy, psychologists should be active in their ‘professional association’ (P-5). The main focus of advocacy is to make ‘policy focus on mental health’ (P-3).

### Particular challenges for Indonesia

Experts were asked to comment on the integration of psychologists into primary health care, specifically in Indonesia. The following are some interesting findings pointed out by experts in the field.

#### Different perspectives on the term ‘generalist’

Psychologists and other health professionals have different expectations about the term psychologist as a *generalist* working in primary health care. The original meaning of *generalist* in the literature is that psychologists need to see patients from different age groups with various disorders (Searight, 2010). But in a practical situation, as a new health professional working in primary health care in Indonesia, psychologists are expected to provide services outside the clinical psychology area of work. For example, one participant reported, ‘In the community, they work at schools. They were expected to measure IQ of students. It's outside their competencies’ (P-20). Another participant explained:

I actually don't want to do something outside clinical psychology competencies, such as to do something related to human resources management. Every year the Government ask us to increase the number of patient visits; therefore, I don't want to handle any work outside of the clinical psychology scope. (FGD-8)

#### Epidemiology: a missing course from the Indonesian curriculum

Epidemiology is an important knowledge area that Indonesian psychologists are never taught during their training. Epidemiology has become very important to assist psychologists in understanding the context of primary health care in the community. One participant highlighted, ‘Psychologists should understand what epidemiology is, the law of epidemiology, the principles of it. If they don't understand, it will be a big barrier between psychologists and other health professionals’ (P-4).

#### Lack of skills in delivering evidence-based treatment

Psychologists are expected to be able to deliver evidence-based treatment, but they do not receive full training in it. One participant stated, ‘They expect psychologists to provide therapy, which is difficult to receive training in [professional training], due to lack of time. Psychologists need to learn independently outside their formal training’ (P-9). A participant reported that psychologists also have experiences where certain Western techniques will not work with patients who have a very limited educational background: ‘In my primary health-care clinic, I mainly use catharsis technique and supportive technique. Cognitive behavior therapy (CBT) does not work on my clients who have very low education’ (FGD-9).

#### Lack of experience on multidisciplinary collaboration

Most participants said that psychologists do not have multidisciplinary collaboration experience in their training, while other professionals, such as doctors, are trained to understand a multidisciplinary perspective. For example, ‘In fourth year, medical students are being trained to solve a case based on multidisciplinary views, such as nurses’, nutritionists' and midwives' view’ (P-22). Another participant suggested that in training, psychologists should ‘have emphasis not exclusively in psychology clinics but also in many other areas such as the general hospital and primary health care’ (P-1) to help psychologists understand other health professionals' perspectives.

#### Low confidence in diagnosing

Participants expected that the diagnosis that is made should be in line with another health professional's diagnosis. A participant explained his past experience in making a diagnosis with a psychologist, ‘To be honest, from my experiences as a psychiatrist, it was not easy to make a diagnosis with psychologists. I did not know why the diagnosis was different for the same patients’ (P-2). Another participant pointed out another experience, ‘once, the doctor referred a patient to the psychologist with mental health problems, but the psychologist could not diagnose any problems and the patient went home without any treatment’ (P-5). ‘Not all schools of psychology are trained to use ICD or DSM in the same standard’ (P-2), one participant pointed out in her summary.

#### A new profession in a very well-established system

The primary health-care system in Indonesia is strong and well established, with doctors and nurses as the main professionals that work in this system. Psychologists need to understand this context and acknowledge possible barriers to working in this system. This was explained by one participant:

The problem of psychologists working with doctors is that doctors don't have enough respect for psychologists, or doctors ask psychologists to take care of difficult patients. So they don't bother them so much. But it's very hard to have a psychologist who could be some new young woman just out of a program … say to the doctor ‘sorry that's not my job, or you're giving the person the wrong medicine’. So, there's a difficulty for psychologists to work in this setting. And then how the psychologists work with the other special teams. You have nurses who've been there for forty years, and a new psychologist comes in a month that teaches them something new about mental illness, so those are some of barriers that are also a simple fact that the job. (P-23)

Another participant expected that psychologists should understand that the clinical work in Indonesian primary health care is not the only main duty: ‘Clinical work actually is the third priority of primary health-care clinics. The first and main duty is actually to develop healthy communities and educate them to lead healthy lifestyles’ (P-14).

## Discussion

The main finding of this study is that in relation to patients, psychologists should have a wide range of roles in primary health care, ranging from promotive to rehabilitative. As part of these roles, psychologists should have the capabilities to conduct assessment, make a diagnosis and provide evidence-based therapy. In relation to other primary health-care providers, psychologists should acknowledge other disciplines in order to be able to collaborate with them. Psychologists should also be able to provide education about mental health to other health professionals.

In relation to primary health care, as service providers, psychologists should be involved in designing a mental health programme. Understanding the success indicators of primary health care in Indonesia is also very important as a basis of programme development and evaluation.

Psychologists should work closely with communities and be able to provide education and manage stigma about mental health. Therefore, psychologists should have social and communication skills to communicate with a wide audience. In relation to policy, psychologists need to understand health systems to be able to advocate to the Government to prioritise mental health.

The findings also illustrate other health professionals' expectation that psychologists will provide brief therapy. This is in line with the characteristics of primary health-care patients pointed out by Hass and deGruy (2004), who expect brief, directive and practical therapy. Psychologists, then, need to have skills in delivering brief therapies (Searight, 2010).

Most participants also highlighted the importance of understanding pharmacology. Psychologists were expected to be able to explain how to use certain medicines, including the side effects of those medicines and even to suggest certain medical treatments to other health professionals. According to Hass and deGruy (2004), one of the characteristic of primary health-care patients is expecting a pharmaceutical-based treatment.

Another finding is that working with the community is a priority in Indonesian primary health care, as every primary health-care clinic in Indonesia is responsible for certain geographical locations and an average of 30,000 people (Abdullah et al., 2012). Searight (2010) pointed out that certain problems might be particularly prevalent in certain communities, relative to the risk factors and habits of those populations. Therefore, working with the community to reduce risk can influence the number of people who need intensive clinical therapy. To be able to work in the community, psychologists need to learn epidemiology and public health.

Compared with previous studies or experiences from other countries (Derksen, 2009; Elder & Silvers, 2009), this study proved that the integration of psychologists into primary health care faces many barriers. These barriers include unmatched expectations from other health professionals compared with the roles and skills that psychologists currently have.

Basic medical education is really important, as pointed out by participants in this research. Consistent with this finding, Dobbie and Mellor (2008) highlighted that psychologists need to be aware of the biological condition and how these conditions can influence their clients' presentations. Primary health care is dominated by medical culture, which presents a problem because psychologists in Indonesia are educated within the social paradigm background. This finding is also in line with the expectations proposed by Hass and deGruy (2004) that patients in primary health care have both medical and psychological conditions, and can also have comorbidity with other conditions.

This study also pointed out that while psychologists are requested to deliver evidence-based therapy, some psychologists experienced that certain evidence-based Western techniques do not work with patients who have a very limited educational background. This finding is in line with international perspectives that suggest CBT needs to be adjusted across cultures (Hays, 2014; Naeem, Gobbi, Ayub, & Kingdon, 2009).

The major strength of this study is that it involved all stakeholders that have made a significant contribution to the integration of psychologists into primary health care in Indonesia, covering the national and local levels for the District where the integration has been piloted. Participants were experts from a range of disciplines that are actively involved in working together in the primary health-care setting, such as nurses, midwives, dentists, public health practitioners and medical doctors. The participants also included health officers, lecturers from universities who give training to psychologists, representatives from two important professional associations in Indonesia and policy-makers involved in the decisions related to integrating psychologists into primary health care.

One weakness of this study is the possibility of interview bias. Since several participants had had previous interactions with DS, these interactions could influence the way they give information. Subjectivity is also another possible bias in choosing the participants. Since Harry Minas is a well-known mental health expert who is well respected in Indonesia, it probably influenced the willingness of participants to participate in this study.

This research can serve as a background for future research into integrating psychology into primary health care in Indonesia. Evaluation research to monitor the impact of the integration is one possible research domain. The process of developing the psychology workforce is another possible domain of research, as is the process of developing training for psychologists who work in primary health care, which could be part of the workforce development domain. The experience of conducting the integration in multicultural Indonesia could be another important research domain.

## Conclusion

Integration of psychologists into primary health care in Indonesia is still in its infancy. It faces many barriers and misunderstandings from other health professionals. Different expectations from government, universities, other health professionals and psychologists themselves mean that the roles of psychologists are not clearly defined. This study is part of four interrelated studies to inform the development of a curriculum for psychologists working in primary health care in Indonesia (Setiyawati, Blashki, Wraith, Colucci, & Minas, 2013a, 2013b; Setiyawati, Colucci, Blashki, Wraith, & Minas, in press).

## References

[CIT0001] Abdullah A., Hort K., Abidin A. Z., Amin F. M. (2012). How much does it cost to achieve coverage targets for primary healthcare services? A costing model from Aceh, Indonesia. *International Journal of Health Planning and Management*.

[CIT0002] http://www.k4health.org/sites/default/files/laporanNasional%20Riskesdas%202007.pdf.

[CIT0003] Bass J., Poudyal B., Tol W., Murray L., Nadison M., Bolton P. (2012). A controlled trial of problem-solving counseling for war-affected adults in Aceh, Indonesia. *Social Psychiatry & Psychiatric Epidemiology*.

[CIT0004] Bowling A. (2009). *Research methods in health: Investigating health and health services*.

[CIT0005] Braun V., Clarke V. (2006). Using thematic analysis in psychology. *Qualitative Research in Psychology*.

[CIT0006] Cavkaytar A., Ceyhan E., Adiguzel O. C., Uysal H., Garan O. (2012). Investigating education and support needs of families who have children with intellectual disabilities. *Turkish Online Journal of Qualitative Inquiry*.

[CIT0007] Chomienne M.-H., Grenier J., Gaboury I., Hogg W., Ritchie P., Farmanova-Haynes E. (2011). Family doctors and psychologists working together: Doctors’ and patients’ perspectives. *Journal of Evaluation in Clinical Practice*.

[CIT0008] Cohen A. (2008). The primary care management of anxiety and depression: A GP's perspective. *Advances in Psychiatric Treatments*.

[CIT0009] Colucci E. (2013).

[CIT0010] Craven M. A., Bland R. (2013). Depression in primary care: Current and future challenges. *La dépression dans les soins de première ligne: enjeux actuels et futurs*.

[CIT0011] Daftar Puskesmas. (2011). http://ilki-online.org/dokumen/tautan/daftarpuskesmas.pdf.

[CIT0012] Das J., Do Q. T., Friedman J., McKenzie D. (2009). Mental health patterns and consequences: Results from survey data in five developing countries. *World Bank Economic Review*.

[CIT0013] Derksen J. (2009). Primary care psychologists in the Netherlands: 30 years of experience. *Professional Psychology: Research and Practice*.

[CIT0014] DeSantis L., Ugarriza D. N. (2000). The concept of theme as used in qualitative nursing research. *Western Journal of Nursing Research*.

[CIT0015] Dobbie M., Mellor D. (2008). Chronic illness and its impact: Considerations for psychologists. *Psychology, Health & Medicine*.

[CIT0016] Eaton W. W. (2012). *Public mental health*.

[CIT0017] Elder M. Q., Silvers S. A. (2009). The integration of psychology into primary care: Personal perspectives and lessons learned. *Psychological Services*.

[CIT0018] Elser J. L., Weisberg R. B., Sciamanna C. N., Bock B. C., Haas L. J. (2004). Anxiety disorder. *Handbook of primary care psychology*.

[CIT0019] Fisher E. B., Chan J. C. N., Nan H., Sartorius N., Oldenburg B. (2012). Co-occurrence of diabetes and depression: Conceptual considerations for an emerging global health challenge. *Journal of Affective Disorders*.

[CIT0020] Friedman J., Thomas D. (2009). Psychological health before, during, and after an economic crisis: Results from Indonesia, 1993–2000. *World Bank Economic Review*.

[CIT0021] Harnowo P. A. (2013). http://health.detik.com/read/2013/07/31/104440/2319785/763/8-provinsi-di-indonesia-tak-punya-rumah-sakit-jiwa.

[CIT0022] Hass L. J., deGruy F. V., Haas L. J. (2004). Primary care, psychology, and primary care psychology. *Handbook of primary care psychology*.

[CIT0023] Hays P. (2014). An international perspective on the adaptation of CBT across cultures. *Australian Psychologist*.

[CIT0024] Himpunan Psikologi Indonesia. (2001). http://himpsi.org/content/view/44/63/.

[CIT0025] Himpunan Psikologi Indonesia. (2003). http://himpsi.org/content/view/34/61/.

[CIT0026] Himpunan Psikologi Indonesia. (2012). http://www.himpsi.or.id/index.php/info-himpsi/sekilas-himpsi.

[CIT0027] Irmansyah I., Prasetyo Y. A., Minas H. (2009). Human rights of persons with mental illness in Indonesia: More than legislation is needed. *International Journal of Mental Health Systems*.

[CIT0028] Jones E. R. (2013). How behavioral health professionals can shape the future of healthcare teams. *Behavioral Healthcare*.

[CIT0029] Jones L. M., Ghani H. A., Mohanraj A., Morrison S., Smith P., Stube D., Asare J. (2007). Crisis into opportunity: Setting up community mental health services in post-tsunami Aceh. *Asia-Pacific Journal of Public Health/Asia-Pacific Academic Consortium for Public Health*.

[CIT0030] Kitzinger J., Barbour R. S., Kitzinger J., Barbour R. S. (1999). Introduction: The challenge and promise of focus groups. *Developing focus group research: Politics, theory, and practice*.

[CIT0031] Laderman M., Mate K. (2014). Integrating behavioral health into primary care. *Healthcare Executive*.

[CIT0032] Mackinlay C. A., Jelinek G. A., Weiland T. J. (2011). The emergency medicine capacity assessment study: Awareness and application of the Australian curriculum framework for junior doctors in the emergency department. *Focus on Health Professional Education: A Multi-disciplinary Journal*.

[CIT0033] Minas H., Diatri H. (2008). Pasung: Physical restraint and confinement of the mentally ill in the community. *International Journal of Mental Health Systems*.

[CIT0034] Morrissey J. (2013). Mind+matter=health care's new math. *H&HN: Hospitals & Health Networks*.

[CIT0035] Moulding R., Blashki G., Gunn J., Mihalopoulos C., Pirkis J., Naccarella L., Joubert L. (2007). *Optimising the primary mental health care workforce: How can effective psychological treatments for common mental disorders best be delivered in primary care*.

[CIT0036] Naeem F., Gobbi M., Ayub M., Kingdon D. (2009). University students’ views about compatibility of cognitive behaviour therapy (CBT) with their personal, social and religious values (a study from Pakistan. *Mental Health, Religion & Culture*.

[CIT0037] Patel V., Araya R., Chatterjee S., Chisholm D., Cohen A., De Silva M., van Ommeren M. (2007). Treatment and prevention of mental disorders in low-income and middle-income countries. *The Lancet*.

[CIT0038] Patel V., Koschorke M., Prince M., Cottler L. B. (2011). Closing the treatment gap. *Mental health in public health: 100 years*.

[CIT0039] Viora E., Maramis A., Keliat B. A., Prasetiyawan (2006). Mental health model of care programmes after the tsunami in Aceh, Indonesia. *International Review of Psychiatry (Abingdon, England)*.

[CIT0040] Puteh I., Marthoenis M., Minas H. (2011). Aceh free Pasung: Releasing the mentally ill from physical restraint. *International Journal of Mental Health Systems*.

[CIT0041] QSR International. (2012). http://www.qsrinternational.com.

[CIT0042] Rabionet S. E. (2011). How I learned to design and conduct semi-structured interviews: An ongoing and continuous journey. *Qualitative Report*.

[CIT0043] Retnowati S. (2011). http://mgb.ugm.ac.id/media/download/pidato-pengukuhan/Page-27.html.

[CIT0044] Rice P. L., Ezzy D. (1999). *Qualitative research methods: A health focus*.

[CIT0045] Sandelowski M., Barroso J. (2003). Classifying the findings in qualitative studies. *Qualitative Health Research*.

[CIT0046] Sanna L., Stuart A. L., Pasco J. A., Kotowicz M. A., Berk M., Girardi P., Williams L. J. (2013). Physical comorbidities in men with mood and anxiety disorders: A population-based study. *BMC Medicine*.

[CIT0047] Searight H. R. (2010). *Practicing psychology in primary care*.

[CIT0051] Souza R., Bernatsky S., Reyes R., Jong K. D. (2007). Mental health status of vulnerable tsunami-affected communities: A survey in Aceh Province, Indonesia. *Journal of Traumatic Stress*.

[CIT0052] Suryani L. K., Lesmana C. B. J., Tiliopoulos N. (2011). Treating the untreated: Applying a community-based, culturally sensitive psychiatric intervention to confined and physically restrained mentally ill individuals in Bali, Indonesia. *European Archives of Psychiatry and Clinical Neuroscience*.

[CIT0053] UGM M. P. P. (2006). http://psikologi.ugm.ac.id/utama/artikel.php?p=373.

[CIT0054] Vaismoradi M., Turunen H., Bondas T. (2013). Content analysis and thematic analysis: Implications for conducting a qualitative descriptive study. *Nursing & Health Sciences*.

[CIT0055] Yurekli A. (2012). An analysis of curriculum renewal in EAP context. *Journal of Instruction*.

